# Clinical Characteristics of Confirmed Cases of COVID-19 Admitted at Al Nahdha Hospital, Oman: A Cross-Sectional Descriptive Study

**DOI:** 10.7759/cureus.17343

**Published:** 2021-08-21

**Authors:** Saud Al Harthi, Magdi Al Osali, Ruwaida Al Ismaili, Sultan Al Lawati, Bina Kamble, Mustafa Al Shaaibi, Nasser Al Kindi, Salim Al Qasabi, Mohammed Alhinai, Hamad Al Harthi, Thamra S Al Ghafri

**Affiliations:** 1 Medicine, Ministry of Health, Al Nahdha Hospital, Muscat, OMN; 2 Internal Medicine, Ministry of Health, Al Nahdha Hospital, Muscat, OMN; 3 Internal Medicine-Gastroenterology, Ministry of Health, Al Nahdha Hospital, Muscat, OMN; 4 Epidemiology and Public Health, Oman Ministry of Health, Directorate of Health Services, Muscat, OMN

**Keywords:** covid-19, oman, clinical, characteristics, critical care

## Abstract

Background and objective

Coronavirus disease 2019 (COVID-19) has become a public health emergency of international concern. Several characteristics of hospitalised cases, including variations in symptoms as well as radiological and laboratory findings, have been described. However, the exact clinical spectrum of hospitalised patients with COVID-19 in Oman is currently unclear. The objective of this paper was to describe the sociodemographic, clinical, laboratory, and radiological characteristics, as well as the treatment and clinical outcomes of the confirmed cases of COVID-19 at Al Nahdha Hospital, Oman. Additionally, factors associated with the severity of the disease were identified.

Methodology

This was a cross-sectional descriptive study of hospitalised COVID-19 patients. The required data were retrieved from the electronic health information system for the period from 3rd March to 9th May 2020. Information was recorded in a bespoke sheet and exported to SPSS Statistics (IBM, Armonk, NY) for analysis.

Results

A total of 102 admissions were included in this study. The mean age of the cohort was 49.9 (14.7) years. The majority were males (77.5%), and more than half were expatriates (56.9%). Diabetes and hypertension were found in 39.2% and 36.3% of the study population, respectively. Common symptoms encountered were dry cough (78.3%), fever (76.5%), and shortness of breath (SOB) (69.6%). Radiologically, bilateral infiltrations were present in 73.5% of the patients. Only 19 cases required critical care (18.6%), and those were mostly older [54.1 (13.4) years], males (89.5%), and non-nationals (63.2%). Significant factors associated with requiring critical care were symptoms of SOB (89.5% vs 65.1%, p=0.03), diabetes (68.4% vs 32.5%, OR=1.5, p=0.004), chronic artery disease (15.8% vs 3.6%, OR=1.7, p=0.04), and diagnosis of acute respiratory distress symptoms (63.2% vs 6.0%, p<0.001). Additionally, the mean ferritin levels were significantly higher in cases requiring critical care [2350.4 (423.8) vs 795.7 (554.3), p=0.005]. Depending on disease severity, the treatment included antibacterials, antivirals, heparin, and steroids. The utilisation of steroids was significantly higher in the cases requiring critical care (63.2% vs 26.5%, p=0.001). Among cases that required critical care (n=19), nine died (death rate=47.4%).

Conclusions

This study has provided fundamental information about the clinical characteristics of confirmed COVID-19 cases in Oman, including factors associated with the disease's severity. Results from this study can be utilised to update the COVID-19 management guidelines for hospitalised patients.

## Introduction

The World Health Organisation (WHO) has declared coronavirus disease 2019 (COVID-19) as a public health emergency of international concern. As of 12th June 2020, a total of 750,3193 laboratory-confirmed cases of COVID-19 had been reported globally, with 421,289 deaths.

The pathogen that causes COVID-19 was identified in samples of bronchoalveolar lavage fluid from a patient in Wuhan, China [1]. The full-genome sequencing showed that this pathogen/virus is distinct from the beta coronaviruses associated with human severe acute respiratory syndrome coronavirus (SARS-CoV) [2]. It has been reported that bats were the primary source of this virus. However, current evidence suggests that it spread to humans via transmission from wild animals illegally sold at the Huanan Seafood Wholesale Market [3].

Despite mild symptoms from beta coronaviruses, mortality rates have been as high as 10% for respiratory illness caused by the SARS-CoV [4] and 37% for Middle East respiratory syndrome coronavirus (MERS-CoV) [5] over the last 20 years. Recent studies have revealed that symptoms of COVID-19 infections are similar to that of SARS-CoV-2 [2]. However, many factors related to the presentation of COVID-19 disease are not yet fully understood [6]. The clinical manifestations reported in patients include fever, non-productive cough, dyspnoea, fatigue, myalgia, normal or decreased leukocyte counts, and radiographic evidence of pneumonia. Organ dysfunction and death have been reported in severe cases [2].

Like all countries globally, Oman has also responded resolutely to the alert from WHO about the rapid spread of this virus in February 2020. The first case of COVID-19 in Oman was identified on 23rd February 2020, and it was linked to travel from a nearby country. As of 12th June 2020, 21,071 cases were identified with 7,489 recoveries and 96 deaths [7].

The Ministry of Health undertook several interventions in Oman to combat the COVID-19 pandemic. According to the national COVID-19 guidelines for Muscat governorate [7], mild and moderate cases are admitted to the Al Nahdha Hospital. The department of medicine at Al Nahdha Hospital (a secondary care hospital for medical services) in Muscat Governorate receives referred patients from all the primary care centres (government and private) and the hospitals' emergency departments. The department has 40 beds, of which six are considered high-dependency care beds for critically sick cases. Due to the unavailability of intensive care services, patients requiring critical care/ventilators were all moved to other tertiary hospitals: The Royal Hospital or Sultan Qaboos University Hospital (SQUH).

Evidence from China has revealed several characteristics of hospitalised cases of COVID-19, including variations in symptoms as well as radiological and laboratory findings [8]. The exact clinical spectrum of hospitalised patients with COVID-19 in Oman is currently inconclusive. Hence, the aim of this study was to describe the demographic details and clinical, laboratory, radiological, and treatment history of all COVID-19 inpatients at Al Nahdha hospital during the period from 3rd March to 9th May 2020. Additionally, this study has looked into the factors associated with the severity of COVID-19, which is defined as patients requiring critical care (transferred to other tertiary hospitals for intensive care services).

This article has already been published on the preprint server Research Square.

## Materials and methods

This was a cross-sectional descriptive study about confirmed COVID-19 cases at the Al Nahdha Hospital. All admitted adult patients with COVID-19 (confirmed cases) from 3rd March to 9th May 2020 were included. COVID-19 was diagnosed on the basis of the WHO 2020 interim guidelines [6]. A confirmed case of COVID-19 was defined as one with the positive result for real-time reverse-transcriptase-polymerase-chain reaction (RT-PCR) assay of nasal and pharyngeal swab specimens [2]. Only laboratory-confirmed cases were included in the analysis. Data was reviewed and analysed by a research team from the Department of Medicine.

Data sources/measurement

The hospital's health information system, "Al Shifa system", was used as the main source of data. A trained team of clinical researchers reviewed the data. The information recorded included the following parameters:

Demographic data: age, gender, nationality, employment, and referring institute.

Risk factors: history of travel, smoking and alcohol consumption, obesity, medical history of comorbidities [diabetes, hypertension, coronary artery disease (CAD), asthma, chronic kidney disease (CKD), heart failure (HF), and any other chronic diseases].

Symptoms: headache, fever, sneezing, cough, shortness of breath (SOB), sore throat, diarrhoea, loss of smell, loss of taste, body aches, and others.

Laboratory reports: complete blood count (CBC), white blood cells (WBC), c-reactive protein (CRP), erythrocyte sedimentation rate (ESR), D-dimer, troponin T, lactate dehydrogenase (LDH), ferritin, liver function tests [alanine aminotransferase (ALT), aspartate aminotransferase (AST), alkaline phosphatase (ALP), bilirubin, and creatinine].

Radiology reports: chest X-ray (CXR).

Given diagnosis: pneumonia and acute respiratory distress syndrome (ARDS) according to the national clinical management pathways for hospitalised patients with COVID-19 [7].

Treatment measures: antibiotics, antiviral therapy, corticosteroid therapy, respiratory support, and other supportive therapies according to the COVID-19 national guideline.

The hospital stay was calculated from the day of admission to the Al Nahdha Hospital to the day of discharge or transfer to another tertiary care hospital for critical care.

Statistical analysis

Frequency tables [mean and interquartile range (IQR) values] were used to describe continuous variables. The categorical variables were described as frequency rates and percentages. Except for age, duration of symptoms, pre-diagnosis, and hospital stay, all other variables were classified into two categories based on the national guidelines and cut-off points. Chest X-ray was categorised into normal, unilateral, or bilateral infiltrates. The variable on severity was divided into "severe" if patients required critical care or "non-severe" if they did not require critical care. Differences in proportions of "severe" and "non-severe" cases were compared across the studied variables. Average point statistical imputations were used for missing laboratory data. For comparisons of means between two independent continuous samples, a t-test was used when the data were normally distributed. Proportions for categorical variables were compared using the χ2 test, although the Fisher's exact test was used when the data were limited. All statistical analyses were performed using SPSS Statistics version 22 software (IBM, Armonk, NY). A two-sided α of less than 0.05 was considered statistically significant.

## Results

The trend of admissions from 3rd March to 9th May

The first admission of a confirmed COVID-19 case at Al Nahdha Hospital was on 3rd March 2020. The number of admissions has varied from a minimum of one to a maximum of seven per day. At the end of the study period, out of 102 admitted cases of COVID-19, 18.6% (n=19) required critical care and were transferred to other tertiary hospitals as severe cases while 81.4% (n=83) were discharged from Al Nahdha hospital. The mean duration of hospital stay was 6.1 (3.4) days [3.7 (2.1) days for the severe cases vs 6.7 (3.5) days for all other cases; p<0.00]. Notably, out of the 19 severe cases (those who required critical care), nine died (fatality rate=8.8%), and two patients were still under critical care at the end of the data collection for this study.

Demographic data

The mean age of the study population was 49.9 (14.7) years (Table 1). The majority of patients were males [77.5% (n=79)], and more than half were non-nationals [56.9% (n=58)]. Although not statistically significant, the proportion of expatriates requiring critical care was higher than their Omani counterparts (63.2% vs 36.8%, p=0.6). Of note, 46% of the cases were from India (23%) and Bangladesh (22%) vs 43.1% Omanis. Others were from Pakistan (9.0%), Iran (2.0%), and Lebanon (1%). Data on occupation was missing for almost half of the population; however, 36.3% of the population reported as being employed.

Most of the cases were referred from primary healthcare centres (PHC) (65.7%). Other referrals were from the private health facilities (14.7%), quarantine institutions (4.9%), arrivals from the Muscat International Airport (2.0%), and emergency departments (12.8%).

Patients who required critical care were older than those who did not require critical care [mean age of 54.1 (13.4) years vs 48.9 (14.9) years, respectively]; however, this difference was not statistically significant (p=0.1).

**Table 1:** Sociodemographic characteristics, risk factors, and comorbidities among confirmed COVID-19 cases at Al Nahdha Hospital *Significant at α<0.05; odds ratios were estimated when statistically appropriate COVID-19: coronavirus disease 2019; PHC: primary healthcare centre

Variables	Total population (n=102)	Patients not requiring critical care (n=83)	Patients requiring critical care (n=19)	P-value
Sociodemographics				
Age in years, mean (SD), [quartiles]	49.9 (14.7), [36.0, 49,0, 61.3]	48.9 (14.9), [36.0, 48.0, 61,0]	54.1 (13.4), [42.0, 53.0, 66.0]	0.1
Gender, n (%)				0.2
Male	79 (77.5)	62 (74.7)	17 (89.5)	
Female	23 (22.5)	21 (25.3)	2 (10.5)	
Nationality, n (%)				0.6
Omani	44 (43.1)	37 (44.6)	7 (36.8)	
Expatriates	58 (56.9)	46 (55.4)	12 (63.2)	
Occupation, n (%)				0.3
Unemployed	9 (8.8)	9 (10.9)	0	
Employed	37 (36.3)	29 (34.9)	8 (42.1)	
Data missing	56 (54.9)	45 (45.8)	11 (57.9)	
Referred from, n (%)				0.7
PHC	67 (65.7)	55 (66.3)	12 (63.2)	
Non-PHC	35 (34.3)	28 (33.7)	7 (36.8)	
Risk factors				
History of travel, n (%)				0.1
No travel	86 (84.3)	67 (80.7)	19 (100)	
Travel	7 (6.9)	7 (8.4)		
Missing	9 (8.8)	9 (10.8)		
Smoking, n (%)				0.9
No	87 (85.3)	71 (85.5)	16 (84.2)	
Yes	5 (4.9)	4 (4.8)	1 (5.3)	
Alcohol, n (%)				0.7
No	89 (87.3)	73 (88.0)	16 (84.2)	
Yes	3 (2.9)	2 (2.4)	1 (5.3)	
Obesity, n (%)				0.5
No	86 (84.3)	71 (85.5)	15 (78.9)	
Yes	16 (15.7)	12 (14.5)	4 (21.1)	
Comorbidities				
Diabetes (DM), n (%)				0.004* (OR=1.5)
No	62 (60.8)	56 (67.5)	6 (31.6)	
Yes	40 (39.2)	27 (32.5)	13 (68.4)	
Chronic artery disease (CAD), n (%)				0.04* (OR=1.7)
No	96 (94.1)	80 (96.4)	16 (84.2)	
Yes	6 (5.9)	3 (3.6)	3 (15.8)	
Hypertension (HTN), n (%)				0.6
No	65 (63.7)	52 (62.7)	13 (68.4)	
Yes	37 (36.3)	31 (37.3)	6 (31.6)	
Chronic kidney disease (CKD), n (%)				0.9
No	97 (95.1)	79 (95.2)	18 (94.7)	
Yes	5 (4.9)	4 (4.8)	1 (5.3)	
Heart failure (HF), n (%)				0.5
No	99 (97.1)	81 (97.6)	18 (94.7)	
Yes	3 (2.9)	2 (2.4)	1 (5.3)	
Asthma, n (%)				0.6
No	101 (99.0)	82 (98.8)	19 (100)	
Yes	1 (1.0)	1 (1.2)	0	
Old TB, n (%)				0.7
No	101 (99.0)	83 (100)	18 (94.7)	
Yes	1 (1.0)	0	1 (5.3)	
HIV, n (%)				0.8
No	101 (99.0)	82 (98.8)	19 (100)	
Yes	1 (1.0)	1 (1.2)	0	

Risk factors

Most patients had no history of travel to countries with COVID-19 outbreaks (84.3%). The majority of the patients were non-smokers (85.3%) and non-alcohol consumers [89 (87.3%)]. Obesity was reported in only 15.7% of patients, of which only 2% had co-existing diabetes.

Comorbidities

More than a third of the patients had diabetes (39.2%). The proportion of patients with diabetes among those who required critical care was higher than those who did not require critical care (68.4% vs 32.5%), and this difference was statistically significant (p=0.004). Patients with diabetes had higher odds of requiring critical care by 1.5 times than their counterparts without diabetes (Table 1). Similarly, patients with CAD had 1.7 times higher odds of requiring critical care than those who did not have CAD. Further analysis of the CAD sub-group showed that all patients with severe COVID-19 had diabetes. The presence of other comorbidities such as hypertension (36.3%), CKD (4.9%), asthma (1.0%), presence of old tuberculosis (TB) (1.0%), and HIV (1.0%) was all not significantly associated with requiring critical care.

Symptoms

The average number of days from the date of symptom appearance to admission was 7.7 (4.2) days. The most common presenting symptom was SOB (69.6%). The proportion of patients presenting with SOB was significantly higher among those who required critical care compared to those who did not require critical care (89.5% vs 65.1% respectively, OR=1.3, p=0.03) (Table 2).

Differences in proportions between severe cases requiring critical care and non-severe cases across other symptoms like sneezing (15.8% vs 4.8%), dry cough (78.9% vs 78.3%), sputum (31.6% vs 20.5%), chest pain (21.1% vs 25.3%), skin symptoms (only one patient), lethargy (31.6 vs 31.3%), dizziness (5.3% vs 7.2%), headache (21.1% vs 19.3%), fever (68.4% vs 78.3%), diarrhoea (26.3 vs 24.1%), loss of smell (8.4% from those who did not require critical care only), loss of taste (9.6% from those who did not require critical care only), and body aches (15.8% vs 20.5%) were not significant. Significantly (p=0.03), more cases in the non-severe group (27.7%) had sore throat compared to those in the severe group (4.2%) (Table 2).

**Table 2:** Symptoms and diagnosis of the confirmed COVID-19 cases admitted at Al Nahdha Hospital Odds ratios were calculated when statistically appropriate COVID-19: coronavirus disease 2019

Variables	Total population (n=102)	Patients not requiring critical care (n=83)	Patients requiring critical care (n=19)	P-value
Days of symptoms, mean (SD), [quartiles]	7.7 (4.2), [5.0, 7.0, 10.0]	7.6 (4.4), [4.8, 7.0, 10.0]	8.5 (3.3), [6.0, 7.0, 10.5]	0.21
Presenting symptoms				
Sneezing, n (%)				0.09
No	95 (93.1)	79 (92.2)	16 (84.2)	
Yes	7 (6.9)	4 (4.8)	3 (15.8)	
Dry cough, n (%)				0.9
No	22 (21.6)	18 (21.7)	4 (21.6)	
Yes	80 (78.4)	65 (78.3)	15 (78.9)	
Sputum, n (%)				0.2
No	79 (77.5)	66 (79.5)	13 (68.4)	
Yes	23 (22.5)	17 (20.5)	6 (31.6)	
Chest pain, n (%)				0.7
No	77 (75.5)	62 (74.7)	15 (78.9)	
Yes	25 (24.5)	21 (25.3)	4 (21.1)	
Sore throat, n (%)				0.03, OR=3.2
No	78 (76.5)	60 (72.3)	18 (94.7)	
Yes	24 (23.5)	23 (27.7)	1 (5.3)	
Skin symptoms, n (%)				0.6
No	101 (99.0)	82 (98.8)	19 (100)	
Yes	1 (1.0)	1 (1.2)	0	
Lethargy, n (%)				0.9
No	70 (68.6)	57 (68.7)	13 (68.4)	
Yes	32 (31.4)	26 (31.3)	6 (31.6)	
Dizziness, n (%)				0.8
No	95 (93.1)	77 (92.8)	18 (94.7)	
Yes	7 (6.9)	6 (7.2)	1 (5.3)	
Headache, n (%)				0.8
No	82 (80.0)	67 (80.7)	15 (78.9)	
Yes	20 (19.6)	16 (19.3)	4 (21.1)	
Fever, n (%)				0.3
No	24 (23.5)	18 (21.7)	6 (31.6)	
Yes	78 (76.5)	65 (78.3)	13 (68.4)	
Shortness of breath, n (%)				0.03, OR=1.3
No	31 (30.4)	29 (34.9)	2 (10.5)	
Yes	71 (69.6)	54 (65.1)	17 (89.5)	
Diarrhoea, n (%)				0.8
No	77 (75.5)	63 (75.9)	14 (73.7)	
Yes	25 (24.5)	20 (24.1)	5 (26.3)	
Loss of smell, n (%)				0.2
No	95 (93.1)	76 (91.6)	19 (100)	
Yes	7 (6.9)	7 (8.4)		
Loss of taste, n (%)				0.2
No	94 (92.2)	75 (90.4)	19 (100)	
Yes	8 (7.8)	8 (9.6)		
Body ache, n (%)				0.8
No	82 (80.4)	66 (79.5)	16 (84.2)	
Yes	20 (19.6)	17 (20.5)	3 (15.8)	
Given diagnosis				
Pneumonia, n (%)				
No	8 (7.8)	8 (9.6)	0	0.2
Yes	94 (92.2)	75 (90.4)	19 (100)	
Acute respiratory distress syndrome, n (%)				<0.001
No	85 (83.3)	78 (94.0)	7 (36.8)	
Yes	17 (16.7)	5 (6.0)	12 (63.2)	

Given diagnosis

According to the national Omani diagnostic criteria [9], the majority of the patients were diagnosed with pneumonia (92.2%). However, the proportion of patients with ARDS was significantly higher in those requiring vs not requiring critical care (63.2% vs 6.0% respectively, p<0.001) (Table 2).

Laboratory reports

All patients from both groups, requiring and not requiring critical care, had high levels of CRP (100% vs 92.8%), D-dimer (57.9% vs 57.8%), and LDH (94.7% vs 81.9%), respectively. However, these proportions were not significantly associated with the severity of the disease. On the other hand, serum ferritin levels were significantly higher in those requiring critical care (100% vs 80.7%, p=0.03). The results from the t-test for two independent samples consistently showed that the difference in mean ferritin levels between the two groups was significant (p=0.005).

Less than half of the patients had abnormal counts/levels of the CBC parameters [low WBCs (20.6%), low neutrophils (42.2%), and low lymphocytes (43.1%)]. The liver function test showed mixed results. More patients had normal levels of ALT (57.8%), AST (56.9%), ALP (83.3%), and bilirubin (90.2%). Notably, a few patients had high creatinine levels (23.5%), and all blood cultures were negative (Table 3).

**Table 3:** Laboratory and radiological reports of confirmed COVID-19 patients at Al Nahdha Hospital Values formatted in bold and italic signify continuous variables: mean (SD), [IQR] and results from t-test of independent samples COVID-19: coronavirus disease 2019; CRP: c-reactive protein; LDH: lactate dehydrogenase; ALT: alanine aminotransferase; AST: aspartate aminotransferase; ALP: alkaline phosphatase; WBC: white blood cells; CXR: chest X-ray

Variables	Total population (n=102)	Patients not requiring critical care (n=83)	Patients requiring critical care (n=19)	P-value
Laboratory investigation				
CRP (0-5 mg/L)	85.2 (84.7), [22.8, 53.0, 115,0]	79.8 (86.6), [18.0, 45,0, 112.0]	109.1 (72.8), [42.0, 82.0, 151.0]	0.2
Abnormal	96 (94.1)	77 (92.8)	19 (100)	0.2
Normal	6 (5.9)	6 (7.2)		
D-dimer (0.1-0.5 mg/L)	3.2 (15.1), [0.3, 0.6, 1.0]	2.8 (15.1), [0.3, 0.0.6, 0.9]	4.7 (15.2), [0.3, 0.5, 2.2]	0.6
Abnormal	59 (57.8)	48 (57.8)	11 (57.9)	0.9
Normal	43 (42.2)	35 (42.2)	8 (42.1)	
Troponin (0-14 ng/L)	46.6 (275.0), [6.0, 7.0, 13.0]	9.0 (6.9), [5.0, 6.0, 10.0]	182.5 (588.5), [9.5, 12.0, 29.50]	0.3
Abnormal	34 (33.3)	28 (33.7)	6 (31.6)	0.8
Normal	68 (66.7)	55 (66.3)	13 (68.4)	
LDH: Male (135-225 U/L), Female (135-214 U/L)	309.1 (108.7), [232.3, 299.0, 367.8]	289.5 (92.5), [221.5, 292.5, 34.8.0]	392.3 (134.3), [279.5, 384.5, 488.8]	0.06
Abnormal	86 (84.3)	68 (81.9)	18 (94.7)	0.1
Normal	16 (15.7)	15 (18.1)	1 (5.3)	
Ferritin (30-400 μg/L)	21 (2000), [492.5, 814.0, 1240.0]	795.7 (554.3), [372.0, 705.0, 1098.5]	2350.4 (423.8), [702.7, 1288, 1965.5]	0.005
Abnormal	86 (84.3)	67 (80.7)	19 (100)	0.03
Normal	16 (15.7)	16 (19.3)	0	
ALT (4-40 U/L)				0.6
Abnormal	43 (42.2)	36 (43.4)	7 (36.8)	
Normal	59 (57.8)	47 (56.6)	12 (63.2)	
AST (10-42 U/L)				0.5
Abnormal	44 (43.1)	37 (44.6)	7 (36.8)	
Normal	58 (56.9)	46 (55.4)	12 (63.2)	
ALP (36-104 U/L)				0.9
Abnormal	17 (16.7)	14 (16.9)	3 (15.8)	
Normal	85 (83.3)	69 (83.1)	16 (84.2)	
Bilirubin (3-10 umol/L)				0.5
Abnormal	10 (9.8)	9 (10.8)	1 (5.3)	
Normal	92 (90.2)	74 (89.2)	18 (94.7)	
Creatinine (62-106 umol/L)				0.7
Abnormal	24 (23.5)	20 (24.1)	4 (21.1)	
Normal	78 (76.5)	63 (75.9)	15 (78.9)	
WBC count (2.4-9.6 10^9^g/L)				0.6
Abnormal	21 (20.6)	18 (21.7)	3 (15.8)	
Normal	81 (79.4)	65 (78.3)	16 (84.2)	
Neutrophil (10^3^/uL)				0.5
Abnormal	43 (42.2)	36 (43.4)	7 (36.8)	
Normal	59 (57.8)	47 (56.6)	12 (63.2)	
Lymphocyte (10^3^/uL)				0.3
Abnormal	44 (43.1)	34 (41.0)	10 (52.6)	
Normal	58 (56.9)	49 (59.0)	9 (47.4)	
CXR				0.05
Normal	12 (11.8)	12 (14.5)	1 (5.3)	
Unilateral infiltrate	15 (14.7)	14 (16.9)	1 (5.3)	
Bilateral infiltrate	75 (73.5)	57 (68.7)	17 (89.5)	

Radiology reports

CXRs showed bilateral infiltrations in 73.5% of the patients (89.5% in patients requiring critical care vs 68.7% in those not requiring critical care). The difference in proportions in reported findings from the CXRs between the two groups was borderline significant (p=0.05).

Treatment measures

Oseltamivir was utilised by 94.1% of the patients (Table 4). Most patients were on multiple treatment regimens according to the COVID-19 national management guidelines [9]. Hydroxychloroquine alone was given to 3.9% of patients. However, 88.2% (n=90) of patients received it in combination with azithromycin. In addition to the combination of hydroxychloroquine and azithromycin, 37.3% of patients received lopinavir/ritonavir (Kaletra), and 38.2% of patients received interferon. All patients had multiple electrocardiogram (ECG) tests and close monitoring of QT interval (QTc) according to the national guidelines on the use of hydroxychloroquine [9]. Only 3.9% of all ECGs showed prolonged QTc for which hydroxychloroquine and or Kaletra were held. The proportion of patients who received interferon were significantly higher among those requiring critical care (89.5% vs 26.5%, p<0.001).

With regard to antibiotic use, patients were given ceftriaxone (79.4%), clarithromycin (26.5%), doxycycline (2.9%), Augmentin (15.7%), and Tazocin (36.3%). Augmentin was only utilised in patients who did not require critical care (19.3%). The proportion of patients who received Tazocin was significantly higher in those requiring critical care (63.2 vs 30.1%, p=0.02) (Table 4).

Other treatments included the use of heparin in the majority of patients (77.5%). Moreover, steroids were given to 33.3% of patients, significantly higher in those requiring critical care (63.2% v 26.5%, p=0.002) (Table 4).

**Table 4:** Treatment measures among confirmed COVID-19 cases admitted at Al Nahdha Hospital *>96.1% of patients had QTC <490 to monitor the use of hydroxychloroquine; odds ratios were estimated when statistically appropriate LMWH: low-molecular-weight heparin

Treatment	Total population (n=102)	Patients not requiring critical care (n=83)	Patients requiring critical care (n=19)	P-value
Oseltamivir, n (%)				0.2
No	6 (5.9)	6 (7.2)		
Yes	96 (94.1)	77 (92.8)	19 (100)	
Hydroxychloroquine alone*, n (%)				
No	98 (96.1)	80 (96.4)	18 (94.7)	0.8
Yes	4 (3.9)	3 (3.6)	1 (5.3)	
Hydroxychloroquine + azithromycin, n (%)				0.7
No	12 (11.8)	10 (12.0)	2 (10.5)	
Yes	90 (88.2)	73 (88.0)	17 (89.5)	
Kaletra (lopinavir/ritonavir), n (%)				0.1
No	63 (61.8)	61 (73.5)	2 (10.5)	
Yes	39 (38.2)	22 (26.5)	17 (89.5)	
Interferon, n (%)				<0.001, OR=3.5
No	63 (61.8)	61 (73.5)	2 (10.5)	
Yes	39 (38.2)	22 (26.5)	17 (89.5)	
Antibiotics				
Ceftriaxone, n (%)				0.5
No	21 (20.6)	18 (21.7)	3 (15.8)	
Yes	81 (79.4)	65 (78.3)	16 (84.2)	
Clarithromycin, n (%)				0.08
No	75 (73.5)	64 (77.1)	11 (57.9)	
Yes	27 (26.5)	19 (22.9)	8 (42.1)	
Doxycycline, n (%)				0.5
No	99 (97.1)	81 (97.6)	18 (94.7)	
Yes	3 (2.9)	2 (2.4)	1 (5.3)	
Augmentin, n (%)				0.04
No	86 (84.3)	67 (80.7)	19 (100)	
Yes	16 (15.7)	16 (19.3)	0	
Tazocin, n (%)				0.007, OR=3.7
No	65 (63.7)	58 (69.9)	7 (36.8)	
Yes	37 (36.3)	25 (30.1)	12 (63.2)	
Other treatment protocols				
LMWH, n (%)				0.5
No	23 (22.5)	20 (24.1)	3 (15.8)	
Yes	79 (77.5)	63 (75.9)	16 (84.2)	
Steroids, n (%)				0.002, OR=4.7
No	68 (66.7)	61 (73.5)	7 (36.8)	
Yes	34 (33.3)	22 (26.5)	12 (63.2)	

## Discussion

This was a descriptive study that looked into the sociodemographic and clinical characteristics of confirmed cases of COVID-19 at Al Nahdha Hospital in Muscat, Oman. The analysis was confined to admissions from 3rd March to 9th May 2020. Results showed an increase in the number of admissions for confirmed COVID-19 cases in the hospital, reflecting the increase in the total number of confirmed cases nationally. Overall, a total of 102 admissions were included in this study. The mean age of the cohort was 49.9 (14.7) years. The majority of the patients were males (77.5%), and more than half were expatriates (56.9%).

Notably, the population of Oman is 463,1060, of which 41% are non-nationals/expatriates, according to the data from the National Centre for Statistics and Information in 2020. There was a surge of admissions among the expatriates. Hence, the collaboration between the public and private entities was enhanced to ensure Universal Health Coverage (UHC), defined as equity and social justice in providing healthcare services [10,11]. Hence, in Oman, medical services for COVID-19 are free of charge to all expatriates.

Most referrals to Al Nahdha Hospital came from PHCs that are accessible to the public. Risk factors such as a history of travel to a country with COVID-19 epidemic outbreaks, as well as smoking, alcohol consumption, and obesity were all uncommon. However, these results were reported subjectively, indicating the possibility of a recall bias.

Diabetes and hypertension were observed in 39.2% and 36.3% of the study population, respectively. Additionally, the presence of diabetes and CAD in patients in the current study were significantly associated with requiring critical care [12,13]. A meta-analysis from China (n=1527 patients) has reported that the most common cardiovascular-metabolic comorbidities associated with COVID-19 were hypertension, cardio-cerebrovascular disease, and diabetes (17.1%, 16.4%, and 7%, respectively). In this report, patients with diabetes had a 1.5-fold increase in the risk of requiring critical care admission, while those with CAD had a 1.7-fold increase. In a subset of 355 patients with COVID-19 in Italy who succumbed to the disease, the mean number of pre-existing underlying conditions was 2.7, and only three subjects did not have any comorbidities [14].

Like its neighbouring countries (Saudi Arabia, United Arab Emirates, Qatar, and Bahrain), Oman has high rates of diabetes, exceeding the global estimates [15]. Diabetes is consistently reported to be one of the leading causes of morbidity and mortality worldwide [15]. This disease, especially in older populations, is associated with infections, particularly influenza and pneumonia [16]. However, more evidence may be required to explore the factors associated with increased susceptibility to infections in individuals with diabetes and other cardiovascular and renal comorbidities [12,17]. These findings suggest that patients with diabetes and CAD should be protected from COVID-19 and managed promptly if they contract the disease.

Unlike other studies, asthma and other comorbidities in the current study were not significantly associated with requiring critical care [12,13]. Future studies may consider a larger sample size to explore any associations.

Only 19 cases (18.6%) required critical care and thus were transferred to other hospitals with intensive care facilities. Out of the 19 severe cases, 47.4% (n=9) died (8.8% of the total admissions), which is less than reported death rates in similar studies (66.3%) in the UK [18] and more than the reported rate (15%) in China [1,19]. Reasons for these differences in death rates across studies could be attributed to differences in the study populations and hospital capacities.

Notably, out of the commonly reported symptoms of fever, cough, fatigue, and sputum production, this study showed that SOB was the only symptom significantly associated with requiring critical care. The absence of fever in COVID-19 (23.5%) is more frequent than in SARS-CoV and MERS-CoV infections (1% and 2% respectively) [20], and hence afebrile individuals may have been missed if the surveillance case definition was centred around fever detection [21]. Headache, upper respiratory symptoms (e.g., sore throat and rhinorrhea), and gastrointestinal symptoms (e.g., nausea and diarrhoea) occurred less often than reported elsewhere [19]. Although not described in the literature from China, smell and taste disorders (anosmia and dysgeusia) were reported by patients from the current study, confirming the reports from patients with COVID-19 in Italy [14].

In laboratory examination results from several studies, most patients had abnormal or decreased WBC counts, particularly lymphocytopenia [2]. In the current study, less than half of our patients had lymphopenia (43.1%). D-dimer levels were elevated in more than half of the patients. This finding was reported to be high in COVID-19 cases [22]. Serum ferritin was the only parameter that was significantly higher in patients requiring critical care; this is consistent with the findings from a recent study that confirmed high levels of ferritin in severe cases of COVID-19 [23]. Similarly, hyperferritinemia was documented as common in severe cases with septic shock and other medical conditions characterised by macrophage response activation [24]. Thus, high ferritin can be considered as a marker for monitoring the severity of COVID-19 cases.

Abnormalities in CXR were detected in the majority of the patients and were significantly higher in those requiring critical care. This finding confirms the evidence reported in several papers [19]. Additionally, similar to the existing reports, most patients presented with pneumonia and ARDS [19]. In the current study, there was 6% of cases diagnosed with ARDS that did not require critical care. Early management could explain this finding, with supportive care and prone manoeuvres preventing deterioration and thus the need for critical care [25].

Till now, except for meticulous supportive care, there is no specific treatment for coronavirus infection [26]. The approach to this disease is to contain the spread of the virus (use of personal protective equipment and taking precautions to reduce the risk of transmission), early diagnosis, isolation, and supportive treatments for the positive cases [6].

The effectiveness of hydroxychloroquine and antibacterial and antiviral agents are inconclusive [2,27]. Despite the lack of robust evidence on the use of hydroxychloroquine in COVID-19, most of the cases in the current study received it and had favorable outcomes. Further well-designed trials are warranted in the future to evaluate the efficacy of hydroxychloroquine in accelerating the recovery of hospitalised COVID-19 cases.

Most of the patients in this study received antibacterial agents; more than a third received antiviral therapy, 77.5% received heparin, and 33.3% received steroids depending on disease severity. Administrating corticosteroids in severe cases of COVID-19 has been supported in several recent studies from China [28] and the UK [29]. This approach is expected to enhance recovery and reduce mortalities.

This study has some limitations. Firstly, some variables' subjective nature (dates of onset of symptoms, history of smoking, obesity, and other reported risk factors) may have introduced a recall bias. Secondly, this study cohort may represent the more severe end of COVID-19 as asymptomatic cases or those with mild symptoms (treated at home) were not encountered in this study. Thirdly, many patients did not undergo sputum bacteriologic or fungal assessment on admission because medical resources were overwhelmed. Finally, missing data on the cases' socioeconomic status would have highlighted the non-clinical provisional factors associated with the severity of the disease.

Despite these limitations, this study provides evidence on the main sociodemographic and clinical characteristics of confirmed cases of COVID-19 in an Arabic-speaking country where such information is sparse in the current literature. More work is needed specifically from the Arab world to compare and contrast experiences on COVID-19 with the rest of the world.

## Conclusions

Most of the confirmed cases of COVID-19 in this study were males and expatriates. Less than 20% of the study population required critical care. Diabetes was a significant predictor for requiring critical care. Despite some variations in initial symptoms, most COVID-19 patients had respiratory symptoms. Depending on disease severity, the treatment included antibacterials, antivirals, heparin, and steroids. Results from this study can be utilised to design and implement further management plans for COVID-19-positive cases in hospital settings.
